# DOG WALKING MEDIATES THE RELATIONSHIP BETWEEN DOG OWNERSHIP AND NEIGHBORHOOD SOCIAL INTERACTION

**DOI:** 10.1093/geroni/igz038.727

**Published:** 2019-11-08

**Authors:** Katie Potter, Hachem Saddiki, Laura B Balzer

**Affiliations:** 1 University of Massachusetts Dept of Kinesiology, Amherst, Massachusetts, United States; 2 Department of Biostatistics and Epidemiology, University of Massachusetts Amherst, Amherst, Massachusetts, United States

## Abstract

Social interaction may be facilitated by dog ownership. We surveyed 421 pet owners about neighborhood social interactions. Dog owners also completed a dog walking questionnaire. Among adults aged 55+ (n=99; 62.2±5.6 years; 90% female), we tested our hypotheses that (1) dog owners were more likely to meet neighbors than non-dog owners, and (2) increased dog walking frequency was associated with increased neighborhood social interaction. Inverse probability weighting was used to control for differences in age and neighborhood type (rural, suburban/urban) between groups. The probability of meeting neighbors was 2.4x higher (95%CI: 1.5-3.9) for dog than cat owners, after controlling for age and neighborhood type. Among dog owners, the odds of meeting a neighbor were 1.7x higher (95%CI: 0.9-3.1) with each unit increase in dog walking frequency (unit=5walks/week). Our findings suggest that programming to support dog ownership and dog walking among older adults may help reduce social isolation.

